# Factors Affecting Histological Gastric Wall Thickness in Japanese Patients with Obesity

**DOI:** 10.1007/s11695-025-07735-6

**Published:** 2025-02-12

**Authors:** Yuichi Endo, Hiroki Orimoto, Shun Nakamura, Wataru Miyoshino, Yuiko Nagasawa, Yoko Kawano, Hiroomi Takayama, Takashi Masuda, Teijiro Hirashita, Masafumi Inomata

**Affiliations:** Oita University, Ōita, Japan

**Keywords:** Histological gastric wall thickness, Morbid obesity, Laparoscopic sleeve gastrectomy

## Abstract

**Background:**

Laparoscopic sleeve gastrectomy (LSG) has become a widely utilized surgical procedure for losing weight since its approval for insurance coverage in Japan in 2014. Its efficacy has been demonstrated by research, but data concerning gastric wall thickness following surgery remain unavailable. Hence, this study aimed to measure gastric wall thickness in resected, formalin-fixed specimens and explore the influence of obesity-related comorbidities on these measurements.

**Methods:**

This prospective study included 53 patients undergoing bariatric surgery at Oita University Hospital. Full-layer thickness (FLT) and muscle-layer thickness (MLT) in the antrum, body, and fornix of resected gastric specimens were measured. Data on patient demographics, comorbidities, and surgical procedure were also collected and analyzed using JMP software. Furthermore, associations between gastric wall thickness and patient factors were assessed.

**Results:**

The mean FLT in the antrum, body, and fornix was 2.9, 2.6, and 2.3 mm, with corresponding MLT of 1.2, 1.0, and 0.9 mm, respectively. The antrum exhibited the thickest gastric wall, whereas the fornix was the thinnest. Diabetes mellitus (DM) was associated with decreased MLT in the fornix, and obstructive sleep apnea (OSA) affected both FLT and MLT in the antrum.

**Conclusions:**

Comorbidities such as DM and OSA significantly influence gastric wall thickness, particularly in the antrum and fornix. Understanding these variations is critical for optimizing surgical techniques and selecting the right stapler in LSG.

## Introduction

Laparoscopic sleeve gastrectomy (LSG) is the most prevalent surgical procedure for weight loss and metabolic surgery globally [1]. Since its approval for insurance coverage in 2014 in Japan, the number of LSG cases has increased gradually over time [2].

Recent data from Japan have demonstrated the efficacy of sleeve bypass surgery [3–5]. With the recent approval of bariatric surgery for insurance coverage in Japan, this field of medicine is expected to reach a pivotal moment in its development. Both surgical techniques involve stapler use during sleeve gastrectomy, resulting in numerous reports on the optimal stapler choice and the appropriate gastric wall thickness [6–11].

In these reports, gastric wall thickness was quantified by applying a specified amount of pressure and then measuring the anterior and posterior walls of the resected specimen as a single unit using a specialized device [7, 8, 11–14]. However, this method cannot ascertain whether the gastric wall was thickening or thinning. Moreover, no studies have reported histological measurements of the resected specimen. Therefore, we aimed to conduct a histological measurement of the gastric wall thickness in resected formalin-fixed specimens and investigated the factors influencing it.

This study has two principal aims: to ascertain gastric wall thickness in resected formalin-fixed stomachs and to identify the factors influencing this measurement.

## Materials and Methods

### Study Design and Participants

This single-center, prospective study included 53 patients undergoing bariatric surgery (LSG and LSG with duodenojejunal bypass LSG-DJB) at the Oita University Hospital. Table 1 presents the patients’ mean age, preoperative body weight after preoperative weight loss, body mass index (BMI), surgical procedures, and comorbidities. The diagnosis of diabetes mellitus (DM) was made on the basis of a fasting plasma glucose level of ≥ 126 mg/dl or an HbA1c level of ≥ 6.5%. Hypertension was diagnosed as a systolic blood pressure of > 140 mmHg and a diastolic blood pressure of > 90 mmHg, while dyslipidemia was diagnosed as low-density lipoprotein cholesterol, total cholesterol, and triglyceride levels of > 160, > 240, and > 200 mg/dl, respectively, according to the American Society for Metabolic and Bariatric Surgery (ASMBS) outcome reporting standards, and without medication [15]. Preoperative polysomnography was conducted for patients exhibiting clinical symptoms of obstructive sleep apnea (OSA), as delineated by the position statements of the ASMBS for OSA [16]. These symptoms included, but were not limited to, loud snoring, restlessness, apneic periods during sleep, daytime somnolence, feelings of fatigue following sleep, and the ease with which sleep could be initiated. The diagnosis of OSA was made on the basis of an apnea–hypopnea index of ≥ 20/h, indicating continuous positive airway pressure covered by Japanese government health insurance. Furthermore, nonalcoholic fatty liver disease (NAFLD) was diagnosed in patients with a liver-to-spleen ratio of 0.9 on noncontract-enhanced computed tomography images [17].

**Table 1:** Patients’ characteristics

No. of patients	53
Age	44 ± 10
Gender (female/male)	35/18
Preoperative body weight (kg)	102 ± 20
BMI (kg/m^2^)	38 ± 6
Comorbidities	
Diabetes mellitus	24 (46%)
Hypertension	32 (60%)
Dyslipidemia	35 (65%)
Obstructive sleep apnea	27 (52%)
Nonalcoholic fatty liver disease	28 (52%)
Operative procedure	
LSG	50 (94%)
LSG-DJB	3 (6%)

*BMI* body mass index, *LSG* laparoscopic sleeve gastrectomy, *LSG-DJB* laparoscopic sleeve gastrectomy with duodenal-jejunal bypass

Mean ± standard deviation

### Surgical Procedures

The surgical techniques employed for the LSG procedures were those which had been published previously [18]. Briefly, we inserted a flexible endoscope (H260 and Q260; Olympus, Tokyo, Japan) into the stomach and resected the greater curvature portion of the stomach using linear endoscopic staplers approximately 4–5 cm proximally from the pyloric ring toward the angle of His. The stump of the stomach was reinforced using seromuscular interrupted sutures. We used Endo GIA Tri-Staple Technology linear stapler cartridges (Covidien Tri-Staple™, Mansfield, MA, USA) wherein the staples’ height decreases inwards (closed staple height [CSH]: 1.75, 1.50, and 1.25 mm). It is customary to utilize purple cartridges for all stapling procedures prior to the commencement of this study, in which the maximum CSH recorded was 1.75 mm [19]. However, no complications were observed, including bleeding, leakage, stenosis, and adhesive ileus. Consequently, the utilization of purple cartridges was maintained for all subjects in the study.

### LSG-DJB Technique

Generally, after performing the usual LSG, we dissect the duodenum until the gastroduodenal artery is fully visible. We trim the duodenum on the side of the lesser curvature and resect it with a stapler. The right gastric artery is preserved. Next, the small intestine is brought up 2–2.5 m from the ligament of Treitz via the anterior colonic route. A 1.5-cm anastomosis is made between the duodenal end and the elevated jejunum and then sutured in two layers. Finally, Petersens’ defect is closed, and the operation is completed.

### Measurement of Gastric Wall Thickness

**Fig. 1:**
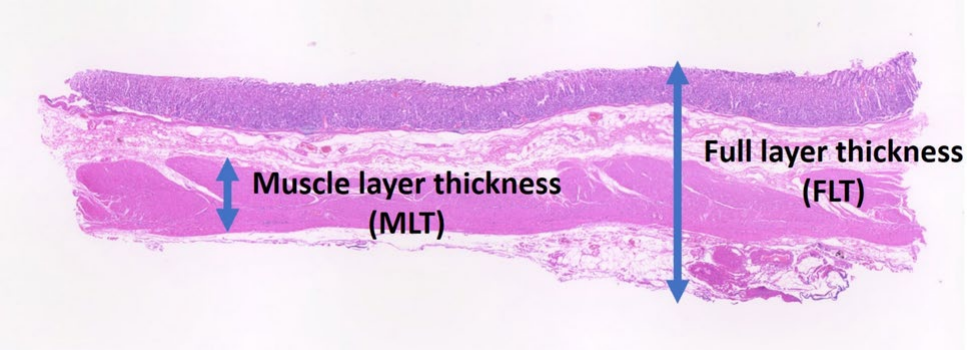
Histological full-layer thickness (FLT) and muscle-layer thickness (MLT)

We measured the histological full-layer thickness (FLT) and muscle-layer thickness (MLT) at the antrum, body, and fornix using resected specimens fixed with formalin. All measurements were taken 1 cm from the staple line to ensure uniformity (Fig. 1).

### Data Collection and Variables

We collected data on patients’ demographic characteristics (age, sex, body weight, and BMI) and comorbidities (DM, hypertension, dyslipidemia, OSA, and NAFLD).

### Statistical Analysis

Data are expressed as mean ± standard deviation. The Mann–Whitney *U* test and Fisher’s exact test were used to analyze continuous and categorical variables, respectively. The GWT results were analyzed using a one-way analysis of variance with Bonferroni correction for multiple comparisons. Finally, Spearman’s rank correlation was used to evaluate the associations between GWT and patient characteristics. A *P*-value < 0.05 was considered statistically significant for all analyses.

All statistical data were analyzed using JMP Pro software version 17 (SAS Institute, Cary, NC, USA).

### Ethical Considerations

This study was approved by the institutional review board, and all patients provided informed consent prior to the study.

## Results

Table 2 presents data on gastric wall thickness measured in three gastric regions: the antrum, body, and fornix. The mean FLT and MLT at the antrum, body, and fornix in the resected gastric specimens were 2.9 and 1.2 mm, 2.6 and 1.0 mm, and 2.3 and 0.9 mm, respectively. For comparison, the results of the device measurements made in the previous study are also presented. The results of the current measurements were similar to those of the previous study [11].

**Table 2:** Data on gastric wall thickness measured in three gastric regions

	Gastric wall thickness (device)	Full-layer thickness (FLT)	Muscle-layer thickness (MLT)	Ratio of muscle (%)
Antrum (mm)	3.1 ± 0.5	2.9 ± 0.7	1.21 ± 0.5	41 ± 10
Body (mm)	2.9 ± 0.5	2.6 ± 0.6	1.0 ± 0.4	38 ± 9
Fornix (mm)	2.0 ± 0.5	2.3 ± 0.6	0.9 ± 0.4	36 ± 8

The ratio of muscle layers was consistent across all regions, indicating a uniform proportion of muscle thickness throughout the stomach. However, the antrum had the thickest gastric wall and muscle layer, with the highest muscle ratio. Conversely, the fornix had the thinnest gastric wall and muscle layer, with the lowest muscle ratio. Both FLT and MLT gradually decreased from the antrum to the fornix, but the MLT gradually decreased from the fornix to the antrum.

Tables 3 and 4 demonstrate the impact of obesity-related comorbidities on both FLT and MLT. In particular, DM significantly affected the MLT in the fornix. OSA and the combination of DM and OSA were associated with a thinner antrum in both FLT and MLT. Moreover, BMI tended to increase thickness, particularly MLT, in the antrum.

**Table 3:** Impact of obesity-related comorbidities on full-layer thickness

	Antrum (FLT)		Body (FLT)		Fornix (FLT)	
	*ρ*	*P*-value	*ρ*	*P*-value	*ρ*	*P*-value
Age	0.0177	0.90	0.0115	0.94	0.0521	0.71
Gender	− 0.1175	0.41	0.1409	0.32	0.0232	0.87
Body weight	0.1488	0.29	− 0.0016	0.99	− 0.1415	0.32
BMI	0.2508	**0.07**	− 0.0430	0.76	− 0.0875	0.57
DM	− 0.0579	0.68	0.0644	0.65	− 0.1004	0.48
HT	− 0.1398	0.32	− 0.1216	0.39	− 0.1608	0.26
OSAS	− 0.3160	**0.02**	− 0.0154	0.91	− 0.0218	0.88
Fatty liver	− 0.0034	0.81	0.1374	0.33	0.0411	0.77
Dyslipidemia	− 0.0391	0.78	0.1093	0.44	0.0715	0.62
DM with OSAS	− 0.2799	**0.04**	− 0.0630	0.65	− 0.0994	0.48

**Table 4:** Impact of obesity-related comorbidities on muscle-layer thickness

	Antrum (MLT)		Body (MLT)		Fornix (MLT)	
	*ρ*	*P*-value	*ρ*	*P*-value	*ρ*	*P*-value
Age	− 0.0461	0.75	− 0.0461	0.79	0.0197	0.89
Gender	− 0.1859	0.19	− 0.0602	0.67	− 0.0219	0.88
Body weight	0.1367	0.33	− 0.0246	0.86	0.0138	0.93
BMI	0.2709	**0.05**	0.0525	0.71	0.0850	0.55
DM	− 0.0039	0.98	− 0.0631	0.66	− 0.3076	**0.03**
HT	0.0588	0.68	− 0.0824	0.56	− 0.0314	0.83
OSAS	− 0.2469	**0.06**	− 0.099	0.49	− 0.0834	0.56
Fatty liver	0.0372	0.79	0.108	0.45	0.0526	0.71
Dyslipidemia	− 0.2035	0.15	0.027	0.85	0.1227	0.39
DM with OSAS	− 0.2716	**0.05**	− 0.1598	0.25	− 0.2128	0.13

## Discussion

In this study, gastric wall thickness (both FLT and MLT) was measured in three regions, namely, the antrum, body, and fornix. The antrum exhibited the greatest thickness, whereas the fornix demonstrated the lowest. Furthermore, obesity-related comorbidities, such as DM and OSA, significantly influenced FLT and MLT, particularly in the antrum and fornix.

Research on gastric wall thickness in patients undergoing sleeve gastrectomy shows significant variation between different gastric regions. The antrum is consistently found to be the thickest, followed by the body, with the fundus being the thinnest [12, 20, 21]. This thickness gradient is critical in selecting appropriate staple sizes to minimize leakage risk [14]. Factors influencing gastric wall thickness reportedly include age, sex, and preoperative weight loss, with males generally having thicker gastric walls [11, 21]. However, gastric wall thickness has not been found to correlate with BMI or comorbidities [20]. Measurement techniques also vary between studies, with different compression pressures applied [14, 21]. Understanding these variations is essential for surgeons to select the correct staple size, thereby potentially reducing the risk of complications in sleeve gastrectomy procedures. All of these studies directly measured the resected stomach, and the thickness was the total of the anterior and posterior walls of the stomach. Therefore, the thickened part of the stomach wall cannot be specified. The present study is the first to measure a formalin-fixed stomach wall specimen histologically.

It has been documented that DM has an impact on the thickness of the gastric wall. In rats with DM, the gastric mucosa thickness has been observed to increase in comparison with that observed in non-DM controls [22]. Histopathological analysis of patients with DM exhibiting severe gastroparesis revealed prominent collagenization and smooth muscle atrophy of the muscle layer in the stomach [23]. Thus, in the presence of DM, the muscular layer may become thinner, but the gastric mucosa may become thicker; consequently, the thickness of the entire gastric wall may not change significantly. These results are also consistent with our results.

The condition of OSA is characterized by repetitive upper airway collapse, resulting in apnea and hypopnea. These occur during sleep, leading to recurrent hypoxia. The consequence of this is fragmented sleep and intermittent drops in arterial blood oxygen saturation (hypoxemia). OSA is most commonly associated with obesity [24], increased cardiovascular risk [25], dyslipidemia [26], DM [27], and liver damage [28]. It is also related to various skeletal muscle alterations. Patients with OSA exhibit lower aerobic capacity and higher muscle microvascularization than controls [29]. The condition of OSA has been shown to be associated with chronic intermittent hypoxia, hypercapnia, and sleep fragmentation [30]. These factors have the potential to affect the structure and function of the upper airway dilator muscles, creating a vicious cycle that exacerbates the condition. In an animal model of OSA, pharyngeal dilator muscles show myopathic changes, including an increased proportion of fast-twitch fibers, fibrosis, and morphologically abnormal fibers, impairing their ability to maintain airway patency [31]. Nevertheless, studies examining the potential relationship between OSA and gastric musculature are still limited. Consequently, the precise impact of this association remains uncertain.

The findings of this study indicate that obesity-related comorbidities have an impact on gastric wall thickness. A reduction in MLT may result in a decline in peristalsis and an increased risk of muscle rupture caused by sudden compression. Sufficient time is essential for both compression and firing.

Of note, this study has several limitations. First, it is a retrospective analysis conducted at a single institution with a relatively small sample size. Second, the impact of compression was not evaluated. Compression is a fundamental aspect of stapling procedures; however, the cross section in this study is difficult to ascertain. However, research employing histological techniques to quantify the thickness of the gastric wall in bariatric surgery cases remains scarce. This information is crucial for future research on gastric wall thickness and the advancement of stapler technology.

## Conclusion

Comorbidities such as DM and OSA may exert a notable influence on gastric wall thickness, particularly in the antrum and fornix. Understanding these variations is crucial to optimize surgical techniques and select the most suitable staplers for LSG.

## Data Availability

No datasets were generated or analysed during the current study.
